# 333. Comparative Efficacy of Rifamycin-containing Regimens in a Murine Model of Tuberculous Meningitis

**DOI:** 10.1093/ofid/ofaf695.116

**Published:** 2026-01-11

**Authors:** Xueyi Chen, Carlos E Ruiz-Gonzalez, Yuderleys Masias-Leon, Medha Singh, Oscar J Nino-Meza, Dmitri Artemov, Charles Peloquin, Sanjay K Jain

**Affiliations:** Johns Hopkins University School of Medicine, Baltimore, MD; Johns Hopkins University School of Medicine, Baltimore, MD; Johns Hopkins University School of Medicine, Baltimore, Maryland, United States., Baltimore, MD; Johns Hopkins University, Baltimore, MD; Johns Hopkins University School of Medicine, Baltimore, MD; Johns Hopkins University School of Medicine, Baltimore, MD; University of Florida College of Pharmacy, Gainesville, Florida; Johns Hopkins University School of Medicine, Baltimore, MD

## Abstract

**Background:**

Tuberculous meningitis (TB meningitis), the most severe form of tuberculosis (TB), is associated with high mortality and neurological sequelae. Standard therapy includes rifampin, a key drug for pulmonary TB; however, its efficacy in TB meningitis is hampered by poor blood-brain barrier penetration, often resulting in subtherapeutic concentrations. While alternative rifamycins like rifapentine and rifabutin exhibit distinct pharmacokinetic advantages, including reduced cytochrome P450 induction, and have been evaluated for pulmonary TB, their utility in TB meningitis remains unexplored.

**Figure 1.**
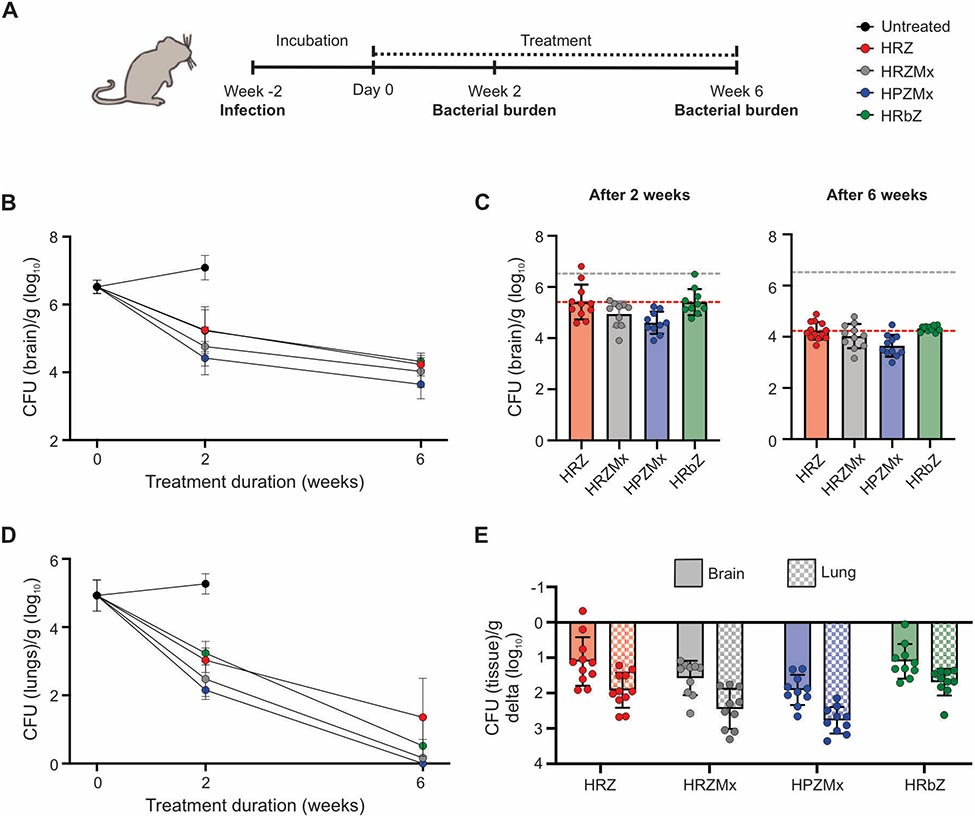
Evaluation of rifamycin-containing regimens in the mouse model of TB meningitis. (A) Mice were infected intraventricularly via Burr hole using a syringe and stereotaxic instrument, treatment were initiated two weeks after (designated as day 0) and continued for six weeks. Mice were randomly allocated to receive multi-drug regimens HRZ, HRZMx, HPZMx, or HRbZ. Isoniazid (10 mg/kg/day), pyrazinamide (150 mg/kg/day), rifampin (10 mg/kg/day), rifapentine (20 mg/kg/day), rifabutin (10 mg/kg/day), and moxifloxacin (100 mg/kg/day divided twice daily) were administered via oral gavage. Mouse dosing was utilized to match the standard human equipotent dosing: isoniazid (10 mg/kg/day), pyrazinamide (25 mg/kg/day), rifampin (10 mg/kg/day), rifapentine (1200 mg), rifabutin (300 mg), and moxifloxacin (400 mg). All regimens received adjunctive dexamethasone via intraperitoneal injection. After completions of antibiotic treatment, mice were sacrificed, tissue were homogenized and colony forming unit (CFU) were enumerated ex vivo. (B-C) Bacterial burden in brain [per gram of brain tissue (log10) from whole brain] at two and six weeks of treatment (animal numbers, n = 11/RHZ, 10/HRZMx, 10/HPZMx, and 10/HRbZ). (D) Bacteria disseminate to the lungs after the brain infection. Therefore, bactericidal activities of the antibiotic rifamycin-containing regimens in brain and lung tissues of the same animal, two and six weeks after treatment initiation, were assessed. (E) Bactericidal activity in brain and lung from the same animals. Data are shown as the reduction in whole brain (solid bar) and lung (checkered bar) organ CFU [per gram of lung tissue (log10) from whole lungs] two weeks after treatment initiation. Animal studies were approved by the Johns Hopkins Animal Care and Use Committee. Data are presented as mean ± SD on a logarithmic scale. Each dot represents a mouse.

**Figure 2.**
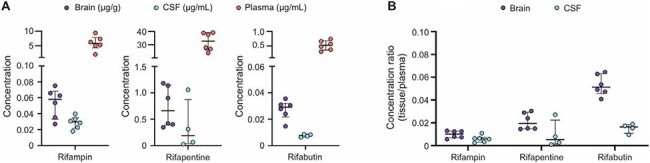
Antibiotic tissue penetration of rifamycins after two weeks of treatment. (A) Brain, CSF and plasma rifampin, rifapentine, and rifabutin levels measured by mass spectrometry in M. tuberculosis-infected mice with TB meningitis after two weeks of treatment. (B) Tissue-to-plasma ratios from brain and CSF tissues for each rifamycin. Rifampin data from six animals; n = 6 brain samples, n = 6 CSF samples, n = 6 plasma samples. Rifapentine data from six animals; n = 6 brain samples, n = 4 CSF samples, n = 6 plasma samples. And Rifabutin data from six animals; n = 6 brain samples, n = 4 CSF samples, n = 6 plasma samples. Data are represented as median ± interquartile range. Each dot represents a mouse.

**Methods:**

We performed preclinical studies in a mouse model of TB meningitis to compare the efficacy and CNS penetration of three rifamycin-containing regimens: standard HRZ (isoniazid, rifampin, pyrazinamide), HPZMx (isoniazid, rifapentine, pyrazinamide, moxifloxacin – recently approved as a 4-month regimen for pulmonary TB), and HRbZ (isoniazid, rifabutin, pyrazinamide). All mice received adjunctive dexamethasone. Mice were intracranially inoculated with *M. tuberculosis* and treatment was started two weeks after incubation. The bacterial burden was quantified after two and six weeks of treatment (Fig. 1A). Drug concentrations in plasma, brain, and cerebrospinal fluid (CSF) were measured by mass spectrometry. Brain magnetic resonance imaging (MRI) with gadolinium-enhanced contrast imaging was performed in *live* animals and postmortem cytokine levels in CSF were used to assess inflammation and tissue damage.

**Figure 3.**
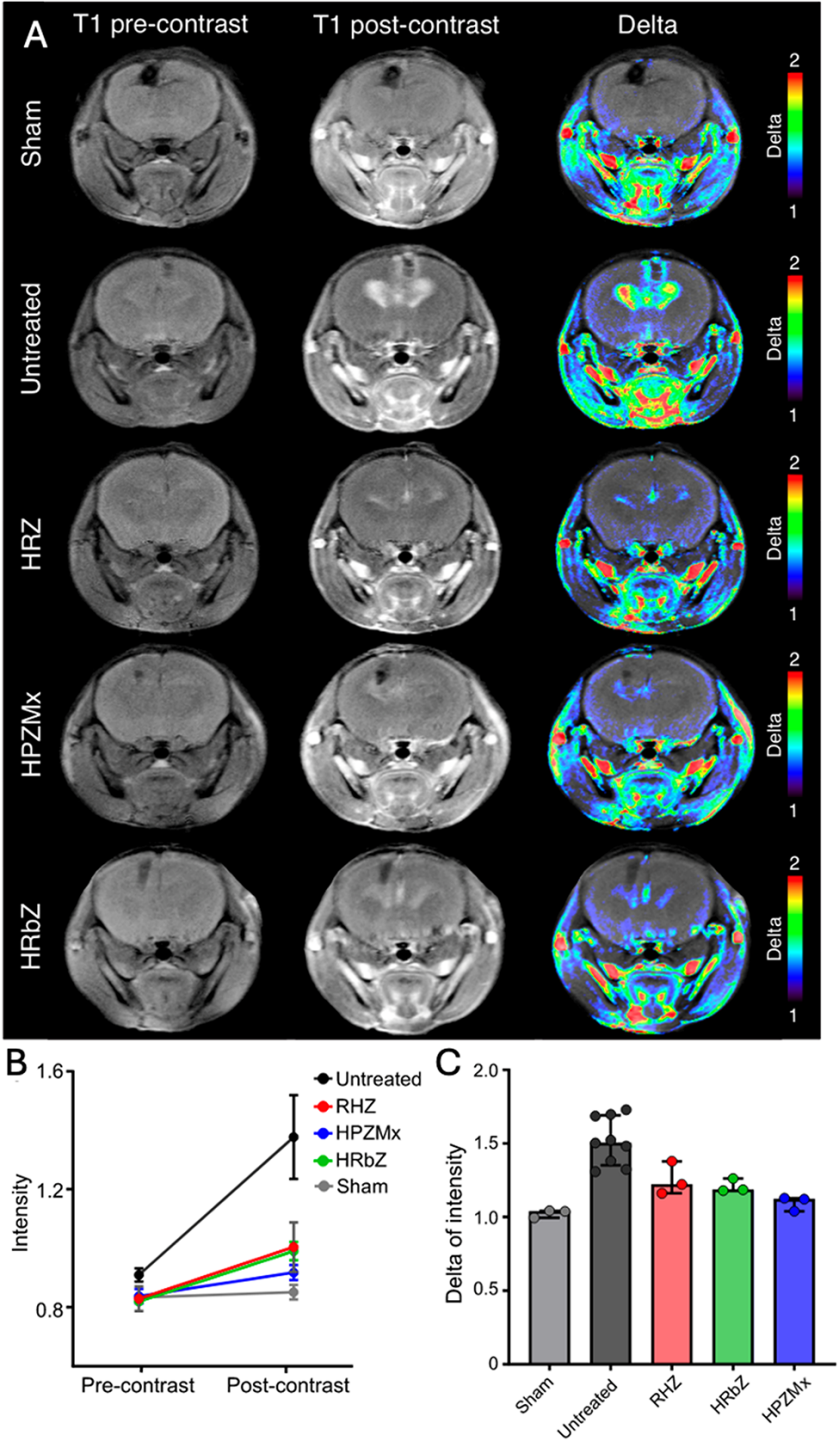
MRI assessment of brain involvement in a mouse model of tuberculous meningitis under different treatment regimens. (A) Representative axial T1-weighted MR images acquired pre-contrast (left column) and post-contrast (middle column) with corresponding delta maps (right column) showing gadolinium contrast enhancement indicative of brain involvement. Images are shown for five groups: Sham (PBS-injected, without infection), untreated (infected, no treatment), and three treatment groups receiving rifamycin-containing regimens-HRZ (isoniazid, rifampin, pyrazinamide), HPZMx (isoniazid, rifapentine, pyrazinamide, moxifloxacin), and HRbZ (isoniazid, rifabutin, pyrazinamide). (B) Quantitative analysis of mean signal intensity before and after contrast administration across groups, showing increased enhancement in untreated mice compared to treated and sham controls. (C) Delta of intensity (post-contrast minus pre-contrast) for each group, demonstrating significant brain involvement in untreated mice and reduced enhancement in all treatment groups. Data are presented as median ± interquartile range. Each dot represents a mouse.

**Results:**

Both rifapentine- and rifabutin-containing regimens demonstrated significant bactericidal activity in the brain, similar to the standard HRZ regimen (Fig. 1B-C). However, activity of all three regimens was higher in the lungs compared to the brain (Fig. 1D-E). All three rifamycins achieved higher concentrations in brain tissue than in CSF (Fig. 2). MRI studies demonstrated substantially reduced tissue damage and inflammation within all regimens (Fig. 3). All regimens also reduced CSF inflammatory cytokine levels.

**Conclusion:**

Rifapentine- and rifabutin-containing regimens demonstrated comparable bactericidal activity to standard HRZ in the brain. These results support further clinical evaluation of rifapentine and rifabutin for TB meningitis.

**Disclosures:**

All Authors: No reported disclosures

